# IBD and Motherhood: A Journey through Conception, Pregnancy and Beyond

**DOI:** 10.3390/jcm12196192

**Published:** 2023-09-25

**Authors:** Antonio M. Caballero-Mateos, Miguel Quesada-Caballero, Guillermo A. Cañadas-De la Fuente, Alberto Caballero-Vázquez, Francisco Contreras-Chova

**Affiliations:** 1 Gastroenterology Unit, Internal Medicine Department, Hospital Santa Ana, 18600 Motril, Spain; ogy1492@hotmail.com; 2 Albayda La Cruz Community Health Center, 18014 Granada, Spain; 3 Brain, Mind and Behaviour Research Center, Faculty of Health Sciences, University of Granada, 18012 Granada, Spain; 4 Pneumology Department, Hospital Virgen de las Nieves, 18014 Granada, Spain; alberto.caballero.sspa@juntadeandalucia.es; 5 Neonatal Care Unit, Pediatrics Department, Hospital Clínico San Cecilio, 18016 Granada, Spain

**Keywords:** inflammatory bowel disease, Crohn’s disease, ulcerative colitis, pregnancy, newborn

## Abstract

Inflammatory Bowel Disease (IBD) presents distinct challenges during pregnancy due to its influence on maternal health and pregnancy outcomes. This literature review aims to dissect the existing scientific evidence on pregnancy in women with IBD and provide evidence-based recommendations for clinical management. A comprehensive search was conducted across scientific databases, selecting clinical studies, systematic reviews, and other pertinent resources. Numerous studies have underscored an increased risk of complications during pregnancy for women with IBD, including preterm birth, low birth weight, neonates small for gestational age, and congenital malformations. Nevertheless, it’s evident that proactive disease management before and throughout pregnancy can mitigate these risks. Continuation of IBD treatment during pregnancy and breastfeeding is deemed safe with agents like thiopurines, anti-TNF, vedolizumab, or ustekinumab. However, there’s a call for caution when combining treatments due to the heightened risk of severe infections in the first year of life. For small molecules, their use is advised against in both scenarios. Effective disease management, minimizing disease activity, and interdisciplinary care are pivotal in attending to women with IBD. The emphasis is placed on the continual assessment of maternal and infant outcomes and an expressed need for further research to enhance the understanding of the ties between IBD and adverse pregnancy outcomes.

## 1. Background

Inflammatory Bowel Disease (IBD) is a long-term condition causing inflammation in the digestive tract. This primarily includes ulcerative colitis (UC) and Crohn’s disease (CD) [1]. The etiology of IBD is multifactorial, encompassing genetic predispositions, aberrant immune responses, alterations in the intestinal microbiota, and environmental influences. Although genetic factors are significant, currently identified genes provide only a partial understanding of disease pathogenesis [2]. Additionally, disruptions in immune regulation and changes in the gut microbiome are also pivotal in the progression of IBD [3,4].

Epidemiological data reveals that the annual incidence of IBD in North America ranges from 0 to 19.2 cases per 100,000 inhabitants while Europe reports an incidence between 0.6 and 24.3 cases per 100,000. The prevalence of IBD is 37.5–248.6 per 100,000 in North America and 4.9–505 per 100,000 in Europe [5]. Historically, the majority of studies pinpointed the peak incidence of IBD in the second to fourth decades of life, a pattern that has remained stable for several decades [5,6]. However, some data sets have indicated a bimodal distribution, with a secondary, albeit modest, peak in the sixth and seventh decades [5]. Notably, these incidence figures have witnessed significant shifts over recent decades [7]. In established populations, the incidence is similar among both men and women, regardless of whether it’s CD or UC [8].

Given the context, IBD is frequently diagnosed during one’s reproductive years, highlighting the need to understand its ramifications on fertility, pregnancy, and childbirth outcomes. As the IBD treatment landscape evolves, introducing new therapeutic avenues over the last decade, novel data exists regarding their safety in pregnancy. In light of these developments, there’s a pressing need for an updated review on IBD’s physiology and management during pregnancy and breastfeeding, as well as the potential implications for the child. This project endeavors to gather and critically analyze the current data, aiming to deepen our understanding of IBD’s impact during pregnancy and to provide evidence-based guidelines for the clinical care of women with IBD who are planning to conceive or are already pregnant.

## 2. Methodology

A comprehensive literature search was conducted on PubMed using keywords: ‘Crohn’s disease’, ‘ulcerative colitis’, ‘IBD’, ‘Inflammatory bowel disease’, ‘pregnancy’, ‘breastfeeding’, ‘birth’, and ‘children’. Additionally, cross-referencing was performed with current treatment modalities. Filters were applied to prioritize studies published in scientific journals, in English, with no date restrictions. Both clinical studies and systematic reviews, meta-analyses, and other relevant resources were considered.

Studies were categorized based on the evidence level, ranging from meta-analyses of clinical trials to opinion articles, reviews, and clinical guidelines. Data tables were constructed highlighting key study details: publication year, authors, study type, subject count, and conclusions.

**Figure 1:**
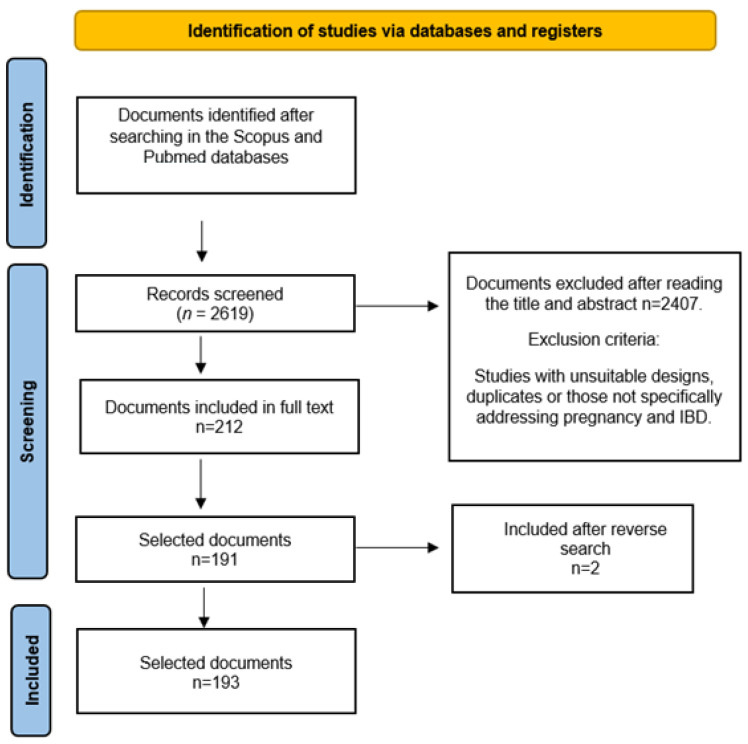
Flowchart.

Study Selection: Inclusion and exclusion criteria were implemented. Inclusion criteria comprised studies assessing IBD’s impact on pregnancy, treatment effects during pregnancy, and pregnancy outcomes for women with IBD, among others. Exclusion criteria encompassed studies with unsuitable designs or those not specifically addressing pregnancy and IBD (Figure 1).

Data Extraction & Analysis: Pertinent data from the selected studies, including sample characteristics, study design, treatments used, pregnancy outcomes, and associated complications, were collated, followed by a descriptive analysis.

## 3. Fertility in IBD Patients

For IBD patients desiring children, fertility is a significant concern. Most studies show that infertility rates in patients with quiescent IBD are similar to those of the general population [9,10,11]. However, active disease has been observed to decrease fertility in both genders [12]. During flare-ups, male IBD patients may experience significantly affected sexual function due to depression, which may interfere with testosterone secretion and spermatogenesis, as well as erectile dysfunction and reduced libido [13,14,15,16]. Regarding fertility in female IBD patients, pregnancy rates have been found to be slightly lower compared to the general population, especially in patients with CD and those who have undergone abdominal surgeries [16,17]. However, women with UC who have not undergone surgery have fertility rates similar to the general population [9,18]. During disease flare-ups, fertility may decrease further, with a live birth rate of 35.6 per 1000 person-years, regardless of the type of IBD, whereas other population studies place it at 49.3 per 1000 person-years [9,19].

Previous surgeries, such as ileo-rectal anastomosis, have been observed to be associated with a significant reduction in fertility rates in UC patients due to the formation of adhesions that could block the fallopian tubes [11,20]. In CD patients, previous abdominal surgeries can also affect fertility [21], although evidence is limited [22].

Family planning is a key aspect for IBD patients. Although fertility itself is not usually affected in these patients, there is a noted decrease in birth rates, which could be due to voluntary decisions by these individuals [9,15]. A reduced interest in having children has been observed, reaching up to 17–37% of patients [23,24]. This decision appears to be associated with various factors, such as fear of a disease relapse, concerns about their medication or the progression of the disease, fear of pregnancy complications, concerns about disease transmission, being over 35, unemployment, being single, or lack of medical counseling [24,25,26]. Likewise, IBD patients who do not have children tend to have less knowledge about fertility, pregnancy, and parity, which has also been related to the lack of medical advice on these topics [27,28]. For this reason, it is important to highlight that proper disease management and appropriate medical guidance before conception can improve reproductive outcomes in IBD patients [29]. Assisted reproduction options, such as in vitro fertilization, which has shown success rates comparable to those in the general population, can be considered for patients with difficulties conceiving [30].

In summary, fertility in IBD patients can be affected by disease activity, abdominal surgeries, and other factors. During flare-ups, both men and women may experience a decrease in sexual function and fertility. A lower willingness to have children has been observed in IBD patients, and previous abdominal surgeries may affect fertility in some cases. Proper disease management and counseling before conception are important for improving reproductive outcomes in these patients. Therefore, it’s crucial to provide them with accurate information and medical advice about fertility and pregnancy. This information could help patients make informed decisions about childbearing and understand how IBD might affect these aspects of their lives.

## 4. Effect of IBD on Pregnancy

The placenta is pivotal in fetal development, managing metabolism, waste disposal, and nutrient exchange through diverse cell types. Placental formation impacts cytokine changes relevant to IBD, with placental macrophages expressing TNF and IL-17—key IBD therapeutic targets [31,32]. Elevated TNF levels in the placenta during pregnancy affect hormone synthesis, placental structure, and embryonic development [33]. Additionally, the placenta’s role in mediating maternal-fetal interactions may have implications for the course of IBD, highlighting its significance in understanding this complex disease context. 

The disease’s activity at the time of conception is the primary factor associated with complications such as premature birth, low birth weight, and being small for gestational age [34,35]. Recent research indicates that the degree of disease activity might play a crucial role in identifying those at risk for adverse outcomes or complications [36,37]. Specifically, only patients who experienced active disease or flare-ups during pregnancy showed higher complication rates [38,39]. Additionally, it’s well-understood that managing IBD activity directly impacts patients’ nutritional status. This impact is evident in pregnancy, where higher complication rates are observed in both the mother and the newborn. In this regard, one study found that weight gain during pregnancy in women with IBD was an indicator of complications during the pregnancy itself [40]. The researchers noted that inadequate weight gain during gestation was associated with active disease presence in both cohorts, which in turn correlated with a higher rate of complications.

## 5. Effect of Pregnancy on IBD

Pregnancy generally has a positive influence on the course of IBD. One study found a 30% risk of disease reactivation during pregnancy, numbers that align with those of non-pregnant women [39]. Yet, more optimistic studies reported a decreased risk of re-lapse compared to the years preceding pregnancy [41]. In this context, a critical determining factor was the state of the disease at the time of conception. Patients who become pregnant during an active disease phase, or shortly after overcoming it, face a higher risk of a flare-up during pregnancy and the postpartum period, compared to those in remission [17,42]. A 2013 meta-analysis reinforced the notion that active UC (RR 2.0, 95% CI 1.5–3) or active CD (RR 2.0, 95% CI 1.2–3.4) at conception is associated with a two-fold in-crease in flare-ups compared to the disease in remission [43]. Because of this, the European Crohn’s and Colitis Organization (ECCO) advises patients to maintain a remission period of more than 6 months before trying to conceive [18].

Finally, it’s essential to underscore that if IBD first manifests during pregnancy in a previously healthy patient, this onset is not linked to a worse prognosis for IBD or for the pregnancy, compared to patients diagnosed before conception [44].

## 6. Monitoring and Management of IBD during Pregnancy

- **IBD Activity Assessment and Biomarkers:** During pregnancy, physiological adaptations can alter the serum biomarkers typically used to evaluate IBD activity. For in-stance, hemoglobin and albumin levels tend to decrease, while C-reactive protein (CRP) and erythrocyte sedimentation rate (ESR) tend to increase [45,46]. Hence, these biomarkers are not viewed as reliable indicators of disease activity during pregnancy. However, fecal calprotectin (FCP), a protein released in response to intestinal inflammation, appears to be a dependable biomarker during pregnancy. Its levels have shown a strong correlation with disease activity, and it can even be suggested as a predictor of symptom onset [47,48,49].
- **Imaging Techniques and Endoscopy:** Intestinal ultrasound has proven useful in determining the activity, extent, and complications of IBD without the need for prior bowel preparation or invasive procedures. Various studies have assessed its feasibility and accuracy in pregnant women with IBD, concluding that it’s an appropriate, non-invasive tool for patient monitoring [27,50]. Magnetic resonance imaging (MRI) can be used without gadolinium, but its utility might be limited in the last trimester due to the interference of fetal structures [51]. Endoscopy during pregnancy poses certain risks, such as an increased chance of premature birth and low birth weight for gestational age [52]. When possible, endoscopy should be avoided, especially during the first trimester. If necessary, rectosigmoidoscopy is preferred over full colonoscopy, and it should be performed without sedation and without bowel preparation [51].
- **Thromboembolic Risk:** Both pregnancy and IBD, especially in its active phase, raise the risk of thromboembolic events (TEV). Thus, it’s essential to assess thromboembolic risk factors before conception and during pregnancy. Starting thromboprophylaxis with low-molecular-weight heparin is recommended for patients admitted due to an IBD flare-up or those undergoing cesarean delivery [53]. Additionally, for patients with a history of TEV or other risk factors, prophylactic anticoagulation should be extended for 3–6 weeks postpartum [54].
- **Surgery in Pregnant Women with IBD:** Indications for surgery in pregnant women with IBD are evaluated similarly as in non-pregnant patients: refractory ulcerative colitis, fulminant colitis, toxic megacolon, or persistent bleeding in UC, and perforation, obstruction, perianal abscess, refractory bleeding, and fulminant colitis in CD [55]. It’s advised to timely perform surgery for appropriate indications instead of waiting for emergent reasons [56].

## 7. Treatments and Pregnancy

Most IBD medications are deemed low-risk during pregnancy [57]. As mentioned, it’s crucial to have the disease well-controlled during pregnancy since IBD flare-ups during this period can increase the risk of obstetric complications. However, due to either lack of awareness or fear of complications, a significant number of patients discontinue maintenance treatment during pregnancy, thereby heightening the risk of relapse [58]. We will detail the treatments used for these diseases and their associated risks, accompanied by a summary table (Table 1):

### 7.1. Steroids

Steroids, often used to treat IBD flare-ups, have been the subject of numerous studies regarding their effects during pregnancy, yielding varied results. Steroids can cross the placenta but are rapidly metabolized into less active metabolites, reducing fetal exposure [55,59,60,61,62]. However, they have been linked to potential adverse effects for both the mother and the fetus, including premature birth, low birth weight, gestational diabetes, and intrauterine infections [63,64,65]. The PIANO (Pregnancy in IBD and Neonatal Outcomes) registry found an increased risk of premature birth, low birth weight, and admission to the neonatal intensive care unit [55]. Older studies suggested a slight increase in the risk of cleft lip [63,64,65], but more recent and extensive research did not confirm this association [66,67]. Rarely, corticosteroids at the end of pregnancy can cause neonatal adrenal suppression, a complication that requires immediate intensive care treatment [68]. Regarding budesonide, a corticosteroid with increased first-pass metabolism and theoretically less fetal exposure [69], although experience is limited, it is considered safe during pregnancy [70,71].

### 7.2. Aminosalicylates (5-ASA)

Aminosalicylates (5-ASA), including mesalamine and sulfasalazine, are generally considered low-risk for use during pregnancy [60]. A meta-analysis demonstrated that the use of 5-ASA compounds during pregnancy is not associated with an increased risk of miscarriage, premature birth, mortality, or congenital anomalies [72]. Mesalamine has limited transplacental transfer and achieves low levels in fetal circulation [73]. Sulfasalazine can affect the absorption of folic acid, so during pregnancy, it should be associated with folic acid supplementation to be safe (>2 mg/day) [74]. On the other hand, in men, sulfasalazine can reduce sperm motility and count and increase abnormal sperm forms [75,76]. Much less frequently, and through unknown mechanisms, the same effect has been observed with mesalamine [77], so it is recommended for men to stop taking sulfasalazine or switch to mesalamine three months before conception [76]. Finally, it’s important to note that the use of mesalamine products is safe during pregnancy despite their presence in umbilical cord blood. No anomalies were observed in the fetuses of 2200 mothers who took up to 3 g of mesalamine daily during pregnancy [72].

### 7.3. Immunomodulators

Thiopurine drugs, such as 6-mercaptopurine and its prodrug azathioprine, are detected in fetal blood, reaching levels of up to 5% of the maternal medication [78]. Older studies suggested that the use of thiopurines during pregnancy led to congenital anomalies, perinatal mortality, low birth weight, infants small for their gestational age, and premature births [79,80]. However, more recent studies and those conducted on transplant patients indicate that the use of thiopurines does not pose an increased risk to fetuses and is safe for continued use in pregnant patients [81,82]. An interim analysis of the PIANO registry confirmed that thiopurine use does not lead to worse fetal outcomes [83].

Methotrexate is an inhibitor of the enzyme dihydrofolate reductase (DHFR), which plays a crucial role in the metabolism of folic acid, which in turn regulates the synthesis of purines and pyrimidines essential for DNA and RNA synthesis [84]. It is currently a drug categorized in pregnancy category X and must be discontinued [85]. This drug has a significant teratogenic effect and has been associated with a high risk of multiple congenital anomalies and severe fetal development complications, including neural tube defects, developmental delay, ileal perforation, abnormal facial features, skeletal deformities, and miscarriages [85,86]. Therefore, its discontinuation is recommended at least 3 months before conception due to its long half-life and persistence in the tissues of the patients who use it, both women and men [87]. Women of childbearing age who take methotrexate should use at least one contraceptive method to prevent pregnancy [84].

Lastly, it’s worth noting that cyclosporine, a calcineurin inhibitor, can be effective in preventing colectomy during severe ulcerative colitis flare-ups during pregnancy [88]. Still, exposure during this period has been associated with maternal hypertension, preeclampsia, miscarriage, premature birth, and low gestational weight, so its use with caution is recommended [89,90].

### 7.4. Anti-TNFa

Anti-TNF drugs, including infliximab, adalimumab, and golimumab, are classified as low-risk in relation to their use during pregnancy for the treatment of IBD [37,91]. These drugs have the ability to cross the placental barrier during the second and third trimesters of pregnancy due to their monoclonal antibody IgG1 structure, without interfering in the organogenesis that occurs during the first trimester [92,93].

The safety of these medications during pregnancy has been studied in-depth. Nu-merous meta-analyses, large-scale retrospective and prospective studies have not shown adverse effects in pregnant women or their offspring with continuous use of anti-TNF medications (Table 2). These studies indicate that exposure to these drugs does not in-crease the rate of congenital anomalies, adverse pregnancy outcomes, or neonatal infections up to the first year of life.

The PIANO study, which included 1490 full-term pregnancies, provided solid data on the safety of anti-TNF therapies in pregnant women with IBD [37]. The study’s results showed that the use of anti-TNF medications did not increase the risk of pregnancy com-plications compared to thiopurines and with the absence of treatment for IBD. Additionally, this study found that the use of thiopurines or anti-TNF was associated with fewer neonatal complications. This finding underscores the importance of maintaining IBD remission during pregnancy.

It is important to note that newborns of mothers who have received infliximab, ada-limumab, or golimumab after week 20 of gestation should not receive live vaccines during their first six months of life because these drugs cross the placenta and can cause immunosuppression in the newborn, given the drug’s persistence detected months after child-birth [94].

In general, the evidence suggests that anti-TNF drugs should continue throughout pregnancy, as a clear increase in IBD flare-ups has been demonstrated with medication interruption [37,91,95]. Therefore, maintaining the mother’s IBD remission is essential, and the decision of when to interrupt anti-TNF therapy should be individualized for each patient [96].

**Table 2:** Anti-TNF treatment studies during pregnancy.

Study	Pregnancies	Live Births (%)	Spontaneous Abortion (%)	Premature Birth	Congenital Anomalies (%)	Low Birth Weight	C-Section
Argüelles et al. [97]	12	12	0	0	0	0	0
Casanova et al. [82]	29	0 (9.1%)	0 (6.1%)	0 (1.7%)	0 (3%)	0	0
Chaparro et al. [91]	388	-	-	10.6%	5.4%	10.6%	43.8%
Correia LM [98]	2	2	0	50% (1)	0	100% (2)	0
Deepak et al. [99]	783	237 (30%)	3.3% (26)	1.7% (13)	1% (8)	9.83% (77)	0
Kane et al. [100]	3	3	0	33% (1)	0	33% (1)	0
Kanis et al. [101]	131	131	6.8% (9)	2.29% (3)	0	0	43.51% (57)
Katz et al. [102]	55	0 (58)	20% (11)	0	0	0	0
Kiely et al. [103]	21	0 (9.52%)	0	0 (2)	0 (9.52%)	0	57.14% (12)
Lichtenstein et al. [104]	162	81 (81.8%)	16 (16.2%)	-	1 (1.2%)	-	-
Mahadevan et al. [83]	10	10	0	30% (3)	0	10% (1)	80% (8)
Moens et al. [105]	186	162	24	14	44	2	-
Schnitzler et al. [106]	35	27 (77.1%)	7 (20%)	17.14% (6)	0	14.28% (5)	0
Seirafi et al. [107]	133	117 (87.96%)	16 (12%)	20% (23)	1% (1)	16% (19)	0
Zelinkova et al. [108]	4	4	0	0	1 (25%)	0	0

### 7.5. Anti-Integrins: Vedolizumab

Vedolizumab is a humanized monoclonal IgG-type antibody that inhibits the binding of α4β7 integrin to the vascular cell adhesion molecule in the mucosa [109]. Like other monoclonal antibodies, it can cross the placental barrier through Fc receptors. Although the information on vedolizumab during pregnancy is more limited compared to anti-TNF drugs. Several studies and retrospective analyses have shown encouraging results. Vedolizumab use has been classified as low risk for use during pregnancy in the treatment of IBD (Table 3). Retrospective analyses in Europe, including the CONCEIVE study (73 patients) and a French study (44 patients), showed no increase in adverse events in patients who received vedolizumab compared to controls on anti-TNF therapy or no therapy [105,110]. Prospective studies in Israel (24 patients) and the United States (41 patients) corroborated these findings [45,111].

However, a 2021 meta-analysis that included four studies in mothers exposed to vedolizumab suggested an increase in the odds of adverse pregnancy outcomes (OR 2.18, 95% CI 1.52–3.13) [112]. But both this study and a previous 2020 vedolizumab safety re-view concluded that disease activity, rather than medication, is likely the main factor in adverse outcomes among patients receiving anti-integrin therapy [113,114].

During pregnancy, maternal elimination of vedolizumab has been shown to increase, so maternal serum levels will decrease [115]. In addition, the placental transfer of vedolizumab is lower than that of anti-TNF agents, resulting in lower drug levels in cord blood at the time of delivery compared to maternal levels [116].

**Table 3:** Studies of Vedolizumab treatment during pregnancy.

Study	Pregnancies	Live Births	Spontaneous Abortions	Congenital Anomalies	Premature Births	Low Birth Weight	C-Section
Moens et al. [105]	24	23	1	3	4	1	5
Bar et al. [111]	24	19	5	1	5	0	0
Mitrova et al. [114]	24	22	2	0	0	1	9
Julsgaard et al. [115]	4	4	0	0	0	0	0
Sheridan et al. [116]	1	0	0	0	0	0	0
Chambers et al. [117]	73	68	12.6%	3 (4.2%)	13.6%	-	-

### 7.6. Anti Il 12/23 and Anti Il 23: Ustekinumab and Risankizumab

Ustekinumab is a human monoclonal antibody that inhibits the activity of the cytokines IL-12 and IL-23, binding to the p40 subunit common to both. Although it has not been extensively studied in pregnant women with IBD, existing data suggest it may be safe (Table 4). The placental transfer pattern of ustekinumab seems similar to that of anti-TNF drugs, with levels in the umbilical cord blood exceeding those in the maternal blood, but without correlation with the interval between the last dose and childbirth [116]. Cases have been reported of mothers who took ustekinumab during pregnancy and lactation without observable negative consequences for them or their children, such as that de-scribed by Klenke et al. [118,119].

Regarding risankizumab, another monoclonal antibody that inhibits the p19 subunit of IL-23, data are even more limited. Although its safety profile is expected to be similar to other biologicals due to its monoclonal antibody structure, a firm recommendation on its safety during pregnancy cannot yet be made [120].

Through various cases, it has been observed that children are born healthy after their mothers were treated with ustekinumab during pregnancy. However, the decision to continue or discontinue treatment during pregnancy must be taken after a careful evaluation of the benefits and risks to the mother and fetus.

**Table 4:** Studies on Ustekinumab Treatment During Pregnancy.

Study	Pregnancies	Live Births	Spontaneous Abortions	Congenital Anomalies	Premature Births	Low Birth Weight	C-Section
Abraham et al. [121]	39	26	8	0	-	3	-
Avni-Biron et al. [122]	27	25	2	1	1	1	10
Chugh R et al. [123]	43	43	0	7	0	1	12
Cortes et al. [124]	1	0	0	0	0	0	0
Galli-Novak et al. [125]	1	0	0	0	0	0	0
Klenske et al. [118]	1	0	0	0	0	0	0
Lukešová et al. [126]	1	0	0	0	0	0	1
Mitrova et al. [114]	32	27	5	3	0	1	8
Rowan et al. [127]	1	0	0	0	0	0	1
Scherl et al. [128]	24	15	4	0	0	0	-
Venturin et al. [129]	1	1	0	0	0	0	0

### 7.7. Small Molecules: JAK and S1P Inhibitors

Tofacitinib, filgotinib, and upadacitinib are inhibitors of Janus kinase (JAK), a type of enzyme involved in various cellular processes. Tofacitinib acts on JAK1 and JAK3, while the latter two are more specific for JAK1. Owing to their small size, they can cross the placenta during the first trimester. Based on animal studies, these drugs aren’t currently recommended during pregnancy. Tofacitinib has been observed to cause teratogenicity in rabbits at levels much higher than the doses used for CU [130]. However, two retrospective reviews of tofacitinib exposure during pregnancy showed no significant adverse outcomes among 11 maternal exposures and 14 paternal exposures in populations with CU and rheumatic diseases [131], and among 47 women with rheumatoid arthritis and psoriasis, 25 healthy newborns, seven spontaneous abortions, eight medical terminations, and only one congenital malformation: pulmonary valve stenosis, were reported [132]. These results seem consistent with background risks in the general population. However, the manufacturer’s recommendations to use effective contraceptive methods during treatment and for 4 to 6 weeks after the last tofacitinib dose should be considered [130].

In terms of men, it’s been shown that tofacitinib doesn’t affect male fertility, sperm quality, sperm motility, or sperm concentration in male rats [130]. Pregnancy outcomes identified from randomized controlled trials of tofacitinib, non-interventional post-approval studies, and spontaneous adverse event reporting did not report fetal deaths or congenital malformations in cases of paternal tofacitinib exposure [131,132]. In summary, while some animal studies raise concerns, human data on Tofacitinib’s impact during pregnancy remains limited, emphasizing the need for caution.

On the other hand, Upadacitinib is a newer agent with limited human safety data. Abbvie Pharmaceuticals has shared exposure data from clinical trials that include 54 total pregnancies. Animal studies revealed a risk of congenital malformations in rats and rabbits at doses similar to those used in humans [133]. It’s recommended that at least 4 weeks elapse between stopping the medication and attempting conception. For both agents, it’s crucial to conduct a risk-benefit discussion with the patient if deciding to continue treatment during pregnancy. Currently, there is no safety data regarding Filgotinib in these circumstances. Given that it’s considered harmful to the fetus, based on animal study findings, both the ECCO guideline and product labeling state that this medication is contraindicated during pregnancy [134].

In the case of ozanimod, a small molecule sphingosine 1-phosphate (S1P) receptor modulator, the data are limited, so its use during pregnancy is not recommended [135]. In animal studies, high doses caused embryofetal mortality in rats and rabbits, and vascular malformations appeared at doses twice that given to humans [136].

## 8. Childbirth

Decisions surrounding childbirth for patients with IBD should be steered by a thorough evaluation of each patient’s unique situation, with an emphasis on both mother and child’s safety. The type of IBD should not exclusively influence the choice of childbirth method. Current clinical guidelines recommend that IBD patients continue their treatments during childbirth unless there’s a complication, like an infection during the peripartum period, prompting a temporary cessation of biologic therapy [137].

It’s noteworthy that cesarean section rates are higher among IBD patients compared to the general population [17,138]. This trend is more pronounced in CD patients than in UC [139]. However, vaginal delivery isn’t linked with an increased incidence of perineal tears in IBD patients, nor does it predispose them to the onset of perianal disease if they were previously unaffected [140]. It’s crucial to highlight that cesarean sections come with their set of complications. A Danish study discovered that IBD patients undergoing cesarean sections had elevated rates of intestinal surgery within 30 days post-partum com-pared to their vaginal birth counterparts [141].

In some scenarios, a cesarean might be advisable, such as for women with active perianal disease or a previous rectovaginal fistula [137]. The presence of an ileo-pouch anal anastomosis (IPAA) can introduce added complexities to the childbirth method decision. Some studies have noted a deterioration in symptoms in CD patients with active perianal disease following vaginal delivery and a decline in the reservoir function in cases of complicated vaginal childbirth [140]. These findings, however, are disputed [137], and the childbirth method choice for IPAA patients should be made judiciously by a multidisciplinary team consisting of obstetricians, surgeons, and gastroenterologists [137].

Given these considerations, the childbirth method selection for IBD patients should be individualized, factoring in disease activity, presence of perianal disease or ileo-anal reservoir, and obstetric indications. Cesarean sections should be reserved for those at heightened risk of perineal tears and obstetric injuries, particularly in cases with active perianal disease or an ileo-anal reservoir [140].

## 9. Breastfeeding

Breastfeeding offers potential advantages over formula feeding. It provides a complete nutritional source and contains immunoglobulins that transfer from the mother to the child, aiding the development of the child’s immune system. Pediatric societies advocate for exclusive breastfeeding for at least the initial six months post-birth and suggest continuing it alongside other foods for at least another six months [142]. However, when choosing to breastfeed for its health benefits to the child, IBD patients also undertake the risk of exposing the infant to medications used for their disease management. In the post-partum period, between 45% and 95% of women initiate breastfeeding [143]. Over time, many women choose to cease breastfeeding due to perceived inadequate milk production and concerns about exposing the baby to drugs through breast milk [144].

Most medications used for IBD are considered breastfeeding-compatible. Even though traces have been found in milk, it doesn’t indicate a biological effect on the new-born. As previously mentioned, methotrexate is contraindicated during breastfeeding [145,146]. Mesalamine and sulfasalamine are excreted in breast milk in minimal amounts, making them compatible with breastfeeding [147]. Prednisone and prednisolone are considered breastfeeding-friendly, but a gap of about 4 h is recommended between drug intake and breastfeeding to reduce drug concentration in the blood [148]. For budesonide, this window isn’t necessary since its levels in milk are nearly undetectable. Despite initial reservations about infant immunosuppression with mothers on thiopurine treatment, long-term prospective studies haven’t found evidence of such effects, leading to these drugs being considered safe with continued use [149]. Cyclosporine is likely compatible with breastfeeding, but its concentration in breast milk can vary. It’s known that the dose the infant receives through breast milk is below 2% of the weight-adjusted dose received by the mother. In this case, monitoring the infant’s blood drug levels is advised [150].

Although breastfeeding isn’t contraindicated for patients on anti-TNF treatments, safety data are limited. Infliximab is detectable in breast milk 48 h post-dose, albeit in very low concentrations, much lower than levels found in patients’ serum. Adalimumab isn’t detectable in breast milk 48 h post-administration [91,151]. Infants of patients on combined anti-TNF + immunomodulator therapy exhibited higher infection rates during their first year, likely due to the combined immunosuppressive effect [152]. For Vedolizumab and Ustekinumab, experiences are limited, but they’re presumably safe for breast-feeding, with low or undetectable doses in milk, considering their molecular structure [37,105]. The PIANO registry analysis indicates that breastfeeding in IBD patients on these described medications wasn’t associated with a heightened risk of infections in the child or a delay in their developmental milestones [92].

Most drugs used for IBD are compatible with breastfeeding since the amounts present in breast milk are minimal and don’t have significant biological effects on the infant (Table 5).

## 10. Children of Mothers with IBD

IBD presents not only challenges for affected individuals but also implications for their offspring. Firstly, the heritability of IBD is significant in family planning. Children of parents with IBD have up to a 10.4% risk of developing it, which increases to 33% if both parents are affected [153,154]. On the other hand, breastfeeding has been observed to be a protective factor against the onset of IBD, reducing the risk by 31% in children of mothers without the disease [155].

Secondly, most mothers with IBD have normal outcomes during pregnancy. However, in mothers with CD, there is a higher risk of premature birth, low birth weight, or being small for gestational age [156,157]. Another study found the same outcomes but only in children of mothers undergoing treatment with steroids [158]. Furthermore, maternal UC was associated with a higher risk of congenital malformations, although of minor severity [159]; however, other studies did not observe this [160]. As previously mentioned, these outcomes might be due to abnormalities in the mother’s nutritional status, disease activity, or adverse effects of the treatment. Between 9% and 18% of women with IBD are at risk of premature birth, compared to the general population (5–9%) [38,161,162]. The risk is high in both UC and CD patients and is especially high in patients with active disease. The risk of a low APGAR score at birth is 1.5 times higher for women with IBD who use steroids to treat active disease compared to the general population [163]. No differences in neurodevelopment have been observed in children of IBD patients in their first 7 years compared to the general population [164]. Regarding the risk of infections, as previously mentioned, intrauterine exposure to thiopurines or anti-TNF in monotherapy does not increase the risk of severe infections during the first 5 years of life. However, combined treatment is associated with a higher risk of severe infections during the first year of life [161]. In light of these findings, it’s imperative for healthcare professionals to provide comprehensive guidance to prospective parents with IBD, ensuring both maternal and child health.

## 11. Conclusions

IBD can affect women of reproductive age aspiring for a safe pregnancy. Effective disease control before conception is vital to improve fertility and ensure a healthy pregnancy, thus reducing potential flare-ups. Continuation of IBD treatments during pregnancy and breastfeeding is generally viewed as safe, emphasizing the importance of thorough disease management for both mother and child.

Elevated risks of complications, such as premature birth, low birth weight, and congenital malformations, have been observed in pregnant women with IBD. However, proactive management can alleviate these risks. 

As the field sees the emergence of new therapies and strategies, ongoing evaluations of maternal and infant outcomes are paramount to understand the IBD-pregnancy outcome relationship better. Effective IBD management before and during pregnancy is essential for optimal reproductive outcomes. Consistent treatment and a team-based care approach are crucial. As research progresses, continuous outcome assessments are vital to navigate the complexities of IBD within the pregnancy context.

## Figures and Tables

**Table 1:** IBD Treatments and Pregnancy.

Mechanism of Action	Medication	Recommendation for Initiation or Short-Term Treatment	Recommendation for Maintenance Treatment
**Aminosalicylates**	Mesalazine and sulfasalazine	Low risk	Low risk
**Corticosteroids**	Budesonide and prednisone	Moderate risk, consider alternatives	Not recommended
**Thiopurines**	6-Mercaptopurine and Azathioprine	Not recommended	Low risk
**Antimetabolites**	Methotrexate	Contraindicated	Contraindicated
**Antibiotics**	Ciprofloxacin, Metronidazole	Low risk	Not recommended
**Calcineurin Inhibitors**	Cyclosporine, Tacrolimus	Low risk	Not recommended
**Anti-TNF**	Adalimumab, golimumab, and Infliximab	Low risk	Low risk
**Anti-integrin**	Vedolizumab	Low risk	Low risk
**Anti-IL-23**	Risankizumab	Probably low risk, limited data	Probably low risk, limited data
**Anti-IL-12/IL-23**	Ustekinumab	Low risk	Low risk
**JAK Inhibitors**	Tofacitinib, Upadacitinib, and Filgotinib	Not recommended	Not recommended
**S1P Inhibitors**	Ozanimod	Not recommended	Not recommended

**Table 5:** Safety of IBD treatments and breastfeeding.

Mechanism of Action	Medication	Breastfeeding Recommendation
**Aminosalicylates**	Mesalamine and Sulfasalazine	Low Risk
**Corticosteroids**	Budesonide and Prednisone	Low Risk
**Thiopurines**	6-Mercaptopurine and Azathioprine	Low Risk
**Antimetabolites**	Methotrexate	Not Recommended
**Calcineurin Inhibitors**	Cyclosporine and Tacrolimus	Low Risk
**Anti-TNF**	Adalimumab and Infliximab	Low Risk
**Anti-integrin**	Vedolizumab	Low Risk
**Antibiotics**	Metronidazole	Not Recommended
	Ciprofloxacin	Low Risk
**Anti-IL-23**	Risankizumab	Low Risk
**Anti-IL-12/IL-23**	Ustekinumab	Low Risk

## Data Availability

Not applicable.
